# A ‘Relay’-Type Drug-Eluting Nerve Guide Conduit: Computational Fluid Dynamics Modeling of the Drug Eluting Efficiency of Various Drug Release Systems

**DOI:** 10.3390/pharmaceutics14020230

**Published:** 2022-01-19

**Authors:** Jiarui Zhou, Sanjairaj Vijayavenkataraman

**Affiliations:** 1 The Vijay Lab, Division of Engineering, New York University Abu Dhabi, Abu Dhabi 129188, United Arab Emirates; jz4301@nyu.edu; 2 Department of Mechanical and Aerospace Engineering, Tandon School of Engineering, New York University, Brooklyn, NY 11201, USA

**Keywords:** nerve guide conduit, NGC, peripheral nerve injury, drug-eluting scaffolds, nerve

## Abstract

Nerve guidance conduits (NGCs) are tubular scaffolds that act as a bridge between the proximal and distal ends of the native nerve to facilitate the nerve regeneration. The application of NGCs is mostly limited to nerve defects less than 3 mm due to the lack of sufficient cells in the lumen. The development of drug-release-system-embedded NGCs has the potential to improve the nerve regeneration performance by providing long-term release of growth factors. However, most of the past works only focused on one type of drug release system, limiting the variation in drug release system types and features. Therefore, in this study, computer-aided design (CAD) models were constructed and Computational Fluid Dynamics (CFD) simulations were carried out to investigate the effect of growth factor transporting efficiency on different drug release systems. To overcome the challenges posed by the current NGCs in treating long nerve gap injuries (>4 cm), a novel ‘relay’ NGC design is first proposed in this paper and has the potential to improve the nerve regeneration performance to next level. The intermediate cavities introduced along the length of the multi-channel NGCs act as a relay to further enhance the cell concentrations or growth factor delivery as well as the regeneration performance. Four different drug release systems, namely, a single-layer microsphere system, a double-layer microsphere system, bulk hydrogel, and hydrogel film, were chosen for the simulation. The results show that the double-layer microsphere system achieves the highest growth factor volume fraction among all the drug release systems. For the single-layer microsphere system, growth factor concentration can be significantly improved by increasing the microsphere quantities and decreasing the diameter and adjacent distance of microspheres. Bulk hydrogel systems hold the lowest growth factor release performance, and the growth factor concentration monotonically increased with the increase of film thickness in the hydrogel film system. Owing to the easy fabrication of hydrogel film and the even distribution of growth factors, the hydrogel film system can be regarded as a strong candidate in drug-eluting NGCs. The use of computational simulations can be regarded as a guideline for the design and application of drug release systems, as well as a promising tool for further nerve tissue engineering study.

## 1. Introduction

Peripheral nerve injuries can result from either systemic disease (e.g., diabetes, Guilain–Barre syndrome, carpal tunnel syndrome) or localized damage (e.g., trauma, sports-related stretching/compression, tumor extirpation). Peripheral nerve injuries can be classified into five stages with increasing severity, starting from a self-restorable local conduction block to a complete transection of the nerve. It is hard for the nerve to self-regenerate when it experiences a complete transection; therefore, an external treatment is required to promote the nerve regeneration. Currently, the treatments used can be classified into three major groups: direct coaptation, grafts, and nerve guidance conduits (NGC). Small nerve gaps (<8 mm) can be simply repaired by direct coaptation, which is the most frequently used clinical treatment owning to its short operation time and complete consistency (e.g., axon size, number, distribution) between both sides of the nerves [1,2,3]. For nerve gaps larger than 8 mm, injured nerve can break due to insufficient elasticity and the exceeded tension can damage the blood flow and impede the nerve regeneration [4]. Therefore, grafts are more suitable for long-gap nerve injuries rather than direct coaptation. Grafts can be divided into allografts and autografts. Autograft involves harvesting a section of the nerve from the patient and then transplanting it directly to the injury site. Sural nerve is commonly used as the autograft nerve because of the fast harvesting operation, sufficient length, and fascicular groups [5]. Although autograft suffers from donor site morbidity, considering its excellent regeneration performance, it remains the first choice for long-gap nerve injuries (>4 cm) and is treated as the gold standard among all the clinical methods [6]. The widespread applications of allograft are limited by donor availability and immune rejection reaction, resulting in the inferior status of allografts compared with autografts [7].

Thus, NGC was developed to overcome the limitations posed by direct coaptation and graft treatments. Normally, an NGC is a tubular structure acting as a bridge to connect both proximal and distal sides of the injured nerve. Owing to the advantages of patient-specific customization, availability, synthetic materials, and cell-laden bioinks, NGC is considered to be a potential alternative for clinical treatment [8,9]. However, current commercially available NGCs mostly target medium nerve gaps (<3 cm) with poorer regeneration performance compared to autograft [10]. Since NGC alone produces unsatisfactory results, changes are required to create a more biomimetic environment by integrating growth factors and cells with the NGC. Growth factors are up-regulated immediately after the peripheral nerve injuries, followed by a gradient decrease after long-term denervation [11,12]. Packing growth factors with NGC can maintain a proper growth factor concentration for a long time, supporting cell proliferation and cell survival [13,14]. Cells cultured in cell-laden NGC can proliferate and migrate to both sides of the injured nerve, leading to faster nerve regeneration. By providing a proper electrical stimulus, regeneration performance can be further improved by regulating cell behaviors (e.g., cell differentiation, cell migration). However, FDA approval is required for the clinical translation of NGC [1]. Most commercially FDA-approved NGCs are made from natural material with superior biocompatibility and biodegradability [15]. Compared with growth-factor-embedded NGC, cell-laden NGC requires a much longer time to receive FDA approval, because the mechanism and potential risk of cell differentiation are not fully understood [16]. Therefore, growth-factor-embedded NGC takes the dominant position, benefiting from the simple clinical translation procedure.

Sustained release of growth factors can be achieved by combining growth factors with biodegradable microspheres and hydrogels, thus promoting nerve regeneration in large peripheral nerve gaps [17,18]. A well-established microsphere delivery system is made of poly(lactic-co-glycolic acid), poly(l-lactic acid) and growth factors [19]. These microspheres were embedded in the inner layer of a single-channel NGC, which was then implanted into a 5 cm nerve defect in a rhesus macaque model for over a year [10]. The results showed a sustained release of growth factor over 50 days, and identical or even superior regeneration performance compared with autograft. In hydrogel drug release systems, permeability, swelling ratio, and degradation rate have great influence on drug release performance [20,21]. Hydrogels are hydrophilic polymers that absorb large amounts of water; therefore, high swelling ratio is more likely to lead to a channel blockage [22]. Degradation rate will lead directly to the formation of fractional bulk release, resulting in the rapid release of drugs [21]. The visco-elastic properties of the hydrogel also play a critical role in the drug release characteristics and more so, if the mode of fabrication is 3D printing, as a proper viscosity of hydrogel is the key index for determining printability [23]. High-viscosity hydrogels might block the nozzle tip, increase the shear stress, or decrease the post-printing cell viability (if cells are also suspended in the drug-containing hydrogel), whereas hydrogels with a low viscosity will lead to poor printability and weak post-printing structural stability [24,25,26]. As hydrogels are highly hydrophilic, different strategies of growth factor release need to be applied in an effort to maintain a sustained release other than a rapid burst release. Depending on the immobilization mechanisms, the strategies can be classified into physical encapsulation, covalent conjunctions and extra cellular matrix-inspired immobilization [27]. Growth factor release profiles vary from a couple of days up to 50 days based on the structure pattern and strategies applied [28,29]. To achieve a uniform nerve regeneration rate across the injury site, it is important to evenly distribute growth factors within the NGC. Given the tiny size of NGC (2–9 mm in diameter, 200 μm wall thickness), the drug release system needs to be optimized to maximize the growth factor concentration under the limited size of microspheres and hydrogels.

In this study, we created a 2D multichannel NGC using computational fluid dynamics (CFD) simulation to examine the transporting efficiency of various growth factor delivery systems, which are single-layer microspheres, double-layer microspheres, bulk hydrogels, and hydrogel films. Growth factor release profile is derived from a published resource [19]; then, the growth factor volume fraction is calculated and used as a criterion to examine the growth factor release performance. For the single-layer microsphere system, microsphere features, including microspheres quantity, diameter, location, and adjacent distance, can be further modified. The aim of this paper is to evaluate the magnitude and distribution of growth factor under different delivery systems by giving either a fixed release time or a constant growth factor releases mass. Finally, the preferred growth factor delivery system is selected to optimize the growth factor release performance in the multichannel NGC. 

## 2. Materials and Methods

### 2.1. 2D Multichannel NGC Model

**Figure 1:**
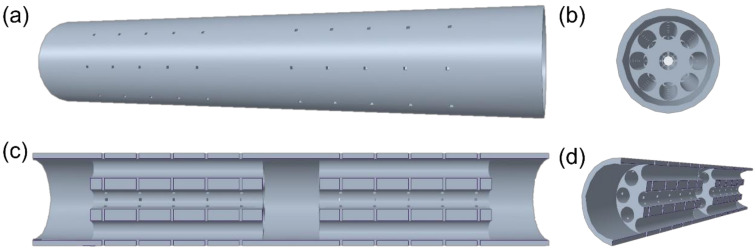
Different views of multichannel NGC: (**a**) standard view; (**b**) side view; (**c**) cross-section view; (**d**) cross-section perspective view.

To achieve a better biomimetic structure, multichannel NGC with 40% porosity was chosen and constructed using ANSYS DesignModeler Geometry (Version 2020 R2) [30]. Each channel corresponds to a single nerve fascicle, which allows parallel nerve regeneration, thereby improving the nerve regeneration efficiency. Figure 1 shows the 3D multichannel NGC models with three internal locations (front, middle and back) available for loading growth factor systems. Based on previous literature, channel diameter and quantities of multichannel NGC can be varied from 200 μm to 660 μm and 4 to 30, respectively [30,31,32,33,34]. Furthermore, the diameter of human sciatic nerve ranges from 2 mm to 9 mm, depending on the location [35]. Therefore, in order to ensure the rationality of the parameters and maintain a suitable porosity (40%), structural features of multichannel NGC including NGC diameter (2 mm), channel diameter (340 μm), and channel quantity (9) were selected in this study. To reduce the simulation complexity, 2D multichannel NGC was chosen and acted as a representative to evaluate the performance of different drug release systems in multichannel NGC. 

### 2.2. Drug Release Systems

**Figure 2:**
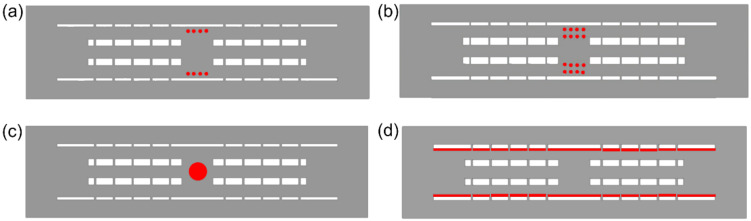
Different types of drug release systems: (**a**) single-layer microsphere system; (**b**) double-layer microsphere system; (**c**) bulk hydrogel system; (**d**) hydrogel film system (grey: fluid region; red: drug release system; white: wall region).

Four types of drug release system were constructed and embedded in the 2D multichannel NGC, namely a single-layer microsphere system, a double-layer microsphere system, growth-factor-embedded bulk hydrogel, and hydrogel film, as shown in Figure 2. Microspheres were fabricated by oil-in-oil emulsion following a 10 min centrifugation, and the sizes of microsphere were mainly between 100 μm and 200 μm [10,19]. For both single- and double-layer microsphere systems, the microspheres had diameters of 100 μm and were placed symmetrically on the upper and lower surfaces of the middle cavity. Microsphere quantities were 8 and 16, respectively, for the two systems, due to the constraint of cavity length. For the single-layer microsphere system, microsphere features including diameter (100–150 μm), quantity (4–8), adjacent distance (0.15–0.25 mm), and locations (front, middle, back) were further modified to examine the growth factor release performance. The default setting of the single-layer microsphere system was 8 microspheres, 0.1 mm diameter, 0.2 mm adjacent distance and middle cavity placement, which served as a standard for all variations of microsphere features. The development of injectable hydrogel enables rapid sol–gel transition time, allowing the formation of hydrogel directly in the middle cavity without breaking the structure into two parts [36]. Considering the inherent biodegradability of hydrogels, a bulk hydrogel diameter of 500 μm was determined, accounting for half of the size of the middle cavity. Apart from bulk hydrogels in the middle cavity, hydrogel can also be coated on the inner surface as a thin layer from the proximal side to the distal side of the multichannel NGC. Growth-factor-embedded hydrogel film has been widely used in skin regeneration and wound healing, with a hydrogel thickness ranging from 30 μm to 1 mm, depending on the specific hydrogel materials used [37,38,39,40,41]. Although there are a variety of available hydrogel thicknesses, the thickness selected in this study was between 100 and 150 μm owing to the limitation of channel size. 

### 2.3. CFD Modeling

The fluid dynamic properties of the 2D multichannel NGC were performed using ANSYS Fluent (Version 2020 R2) under mixture model theory, which has typically been used to simulate particle-laden flows with low loading [42]. The mixture model is designed for multiphase flow, and those phases are considered as interpenetrating continua to simulate the diffusion process. Despite the volume of fraction (VOF) model being known to be the most commonly used approach for multiphase simulation, the design objective of immiscible fluids limits its application in this study. Multiphase flow under the mixture model can be solved by calculating a series momentum, continuity, and energy equations, as listed below. 

The continuity equation for the mixture model is:

(1) $$\frac{\partial }{\partial t}\left(\rho_{m}\right)+\nabla \cdot \left(\rho_{m}{\vec{v}}_{m}\right)=0$$

where ${\vec{v}}_{m}$ is the mass-averaged velocity:

(2) $${\vec{v}}_{m}=\frac{\sum_{k=1}^{n}\alpha_{k}\rho_{k}{\vec{v}}_{k}}{\rho_{m}}$$

and $\rho_{m}$ is the mixture density:

(3) $$\rho_{m}=\overset{n}{\underset{k=1}{\sum }}\alpha_{k}\rho_{k}$$

$\alpha_{k}$ is the volume fraction of phase *k*.

The momentum equation for the mixture model is:

(4) $$\frac{\partial }{\partial t}\left(\rho_{m}{\vec{v}}_{m}\right)+\nabla \cdot \left(\rho_{m}{\vec{v}}_{m}{\vec{v}}_{m}\right)=-\nabla_{p}+\nabla \cdot \left[\mu_{m}\left(\nabla {\vec{v}}_{m}+\nabla {\vec{v}}_{m}{}^{T}\right)\right]+\rho_{m}\vec{g}+\vec{F}+\nabla \cdot \;\left(\sum_{k=1}^{n}\alpha_{k}\rho_{k}{\vec{v}}_{dr,k}{\vec{v}}_{dr,k}\right)$$

where *n* is the number of phases, $\vec{F}$ is body force, and $\mu_{m}$ is the viscosity of the mixture:

(5) $$\mu_{m}=\overset{n}{\underset{k=1}{\sum }}\alpha_{k}\mu_{k}$$

${\vec{v}}_{dr,k}$ is the drift velocity for secondary phase *k*:

(6) $${\vec{v}}_{dr,k}={\vec{v}}_{k}-{\vec{v}}_{m}$$

The energy equation for the mixture model is:

(7) $$\frac{\partial }{\partial t}\overset{n}{\underset{k=1}{\sum }}\left(\alpha_{k}\rho_{k}E_{k}\right)+\nabla \cdot \overset{n}{\underset{k=1}{\sum }}\left(\alpha_{k}{\vec{v}}_{k}\left(\rho_{k}E_{k}+p\right)\right)=\nabla \cdot \left(k_{eff}\nabla T\right)+S_{E}$$

where $k_{eff}$ is the effective conductivity ($\sum \alpha_{k}\left(k_{k}+k_{t}\right)$), $k_{t}$ is the turbulent thermal conductivity. The first term on the right-hand side of equation represents energy transfer due to the conduction. $S_{E}$ includes any other volumetric heat sources.

(8) $$\;E_{k}=h_{k}-\frac{p}{\rho_{k}}+\frac{v_{k}^{2}}{2}$$

for a compressible phase, and $E_{k}=h_{k}$ for an incompressible phase, where $h_{k}$ is the sensible enthalpy for phase *k*.

**Figure 3:**
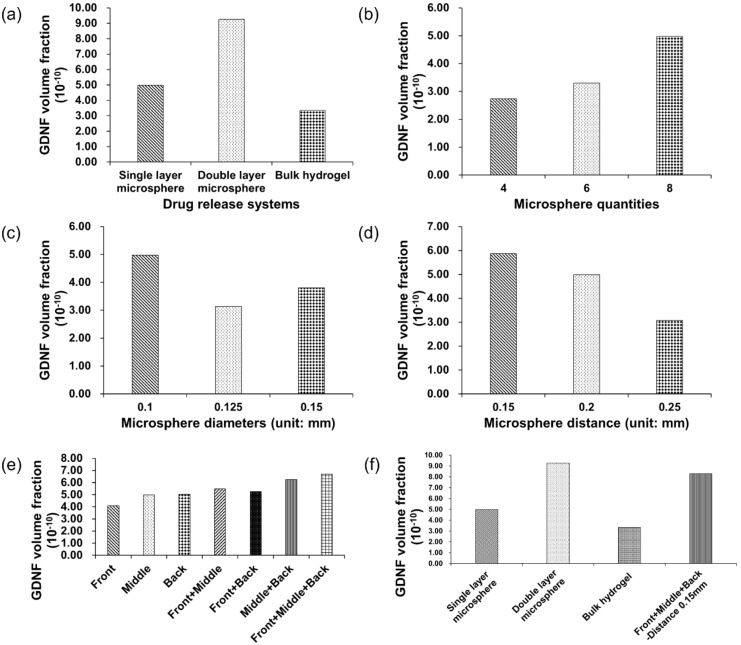
GDNF volume fraction of different drug release systems under the assumption of constant simulation time (519 s). (**a**) Comparison among single- and double-layer microsphere and bulk hydrogel systems. (**b**–**e**) Comparison within single-layer microsphere system only. (**f**) Comparison between three drug release systems and the combined model.

In this work, interstitial fluid and glial cell derived neurotrophic factor (GDNF) were used as two separate phases. Interstitial fluid was chosen to represent the hydrodynamic system around the multichannel NGC, and GDNF has been validated as an effective chemical stimulus to enhance the nerve regeneration performance [43]. Both the phases are treated as Newtonian incompressible fluids, with viscosities of 0.0035 and 0.0015 kg/ms and densities of 1000 and 1370 kg/m^3^, respectively [44,45,46,47]. Since there are no direct data on the properties of GDNF, viscosity and density of GDNF were estimated from the concentration and molecular weight of GDNF, respectively [45,47]. Apart from material properties, four open boundaries were prescribed in the model, including two inlet edges, one outlet edge and a wall boundary at the rest of the edges (Figure 3). Inlet 1 was determined as a fixed velocity equal to the physiological interstitial fluid velocity to better mimic the hydrodynamic system around the multichannel NGC, while the velocity of inlet 2 was composed of a burst release (fast speed) and continuous release (slow speed) based on an earlier validated GDNF release profile [19,46]. A drug release system with reduced release time and cumulative mass was adopted to examine the growth factor release performance while avoiding the excessive computation cost. Given that the release velocity remains the same when scaling down the release time and cumulative mass at the same proportion, the reduced growth factor release profile would still well represent the release performance for the simulation purpose. Table 1 illustrates how to narrow down the original growth factor release profile. 

### 2.4. GDNF Volume Fraction

The magnitude of GDNF volume fraction was directly given by ANSYS Fluent (Version 2020 R2) to evaluate and compare the GDNF release performance among all the drug release systems. To provide a more comprehensive comparison, two control variables were prescribed, which are constant growth factor mass and constant simulation time. Given a constant GDNF density and release rate, growth factor mass is determined by the surface area of the drug release system and simulation time (release time), making the simulation time a dependent parameter of the surface area. Table 2 lists the surface area of each drug release system as well as the corresponding simulation time. The default simulation time is 519 s, as shown in Table 1, with a time step size of 0.5 s. Due to the long and thin structure of the hydrogel film, its surface area is 166 times that of the default single-layer microsphere system. Shortening the simulation time by the same proportion will make the simulation time far less than the unit time step size; therefore, the hydrogel film is not suitable for comparison with other drug release systems. Thus, the growth factor release performance of hydrogel film with different hydrogel film thicknesses would be compared instead of comparing them with the drug release systems.

## 3. Results

### 3.1. A ‘Relay’-Type NGC Design

This study is the first to introduce a ‘relay’-type NGC design, which provides three cavities to load the drug release system, connected by two multichannel NGCs. Some of the biggest factors affecting the NGC nerve regeneration performance are insufficient nerve cell concentrations and sustained nerve growth factor availability NGCs targeting long nerve gap injuries (>4 cm). The intermediate cavities provided could be integrated with drug release systems and culture of nerve related cells [10,48]. Unlike the commonly used multichannel NGC, the extra intermediate cavity of the ‘relay’-type NGC can be used as a relay to further enhance the cell concentration as well as the nerve regeneration performance by loading drug release systems or appropriate cells. Thus, the application of ‘relay’-type NGC has the potential to improve the effective regeneration length of NGC to cater for long nerve gap injuries, which is of huge clinical significance. 

### 3.2. GDNF Volume Fraction under the Assumption of Constant Simulation Time

**Figure 4:**
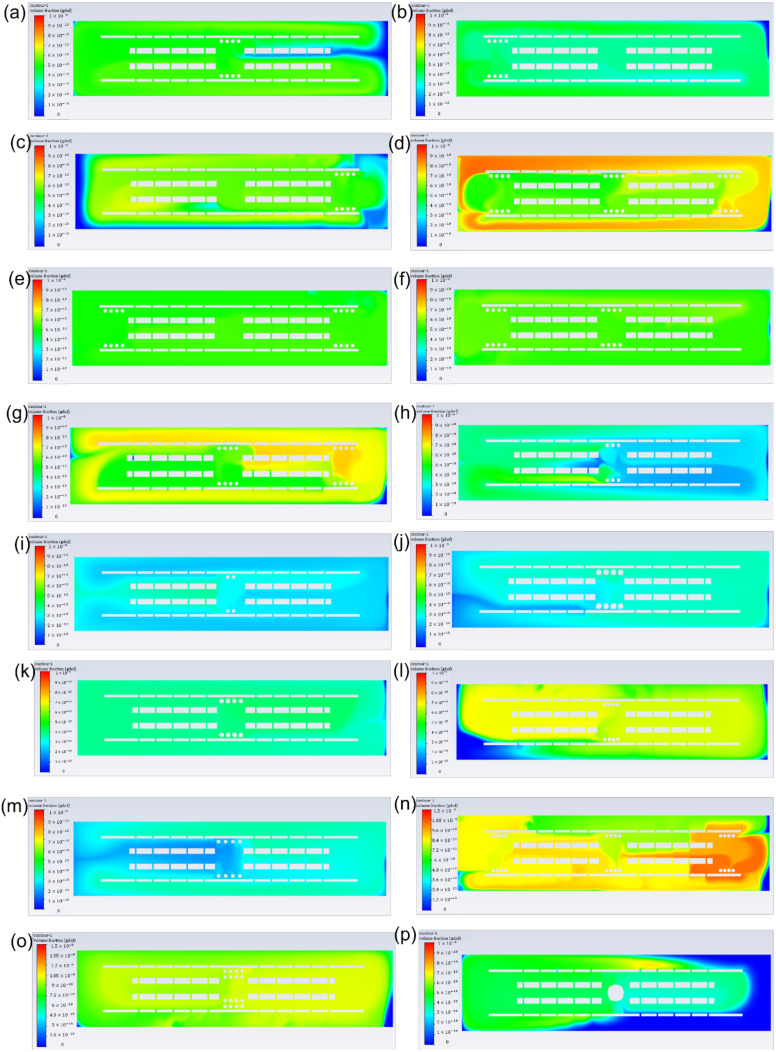
GDNF distribution of different drug release systems under the assumption of constant simulation time. (**a**) Single-layer microsphere system (microsphere features: quantity: 8; diameter: 0.1 mm; adjacent distance: 0.2 mm; position: middle); (**b**) single-layer microsphere system (microsphere features: quantity: 8; diameter: 0.1 mm; adjacent distance: 0.2 mm; position: front); (**c**) single-layer microsphere system (microsphere features: quantity: 8; diameter: 0.1 mm; adjacent distance: 0.2 mm; position: back); (**d**) single-layer microsphere system (microsphere features: quantity: 24; diameter: 0.1 mm; adjacent distance: 0.2 mm; position: front+middle+back); (**e**) single-layer microsphere system (microsphere features: quantity: 16; diameter: 0.1 mm; adjacent distance: 0.2 mm; position: front+back); (**f**) single-layer microsphere system (microsphere features: quantity: 16; diameter: 0.1 mm; adjacent distance: 0.2 mm; position: front+middle); (**g**) single-layer microsphere system (microsphere features: quantity: 16; diameter: 0.1 mm; adjacent distance: 0.2 mm; position: middle+back); (**h**) single-layer microsphere system (microsphere features: quantity: 6; diameter: 0.1 mm; adjacent distance: 0.2 mm; position: middle); (**i**) single-layer microsphere system (microsphere features: quantity: 4; diameter: 0.1 mm; adjacent distance: 0.2 mm; position: middle); (**j**) single-layer microsphere system (microsphere features: quantity: 8; diameter: 0.15 mm; adjacent distance: 0.2 mm; position: middle); (**k**) single-layer microsphere system (microsphere features: quantity: 8; diameter: 0.125 mm; adjacent distance: 0.2 mm; position: middle); (**l**) single-layer microsphere system (microsphere features: quantity: 8; diameter: 0.1 mm; adjacent distance: 0.15 mm; position: middle); (**m**) single-layer microsphere system (microsphere features: quantity: 8; diameter: 0.1 mm; adjacent distance: 0.25 mm; position: middle); (**n**) single-layer microsphere system (microsphere features: quantity: 8; diameter: 0.1 mm; adjacent distance: 0.15 mm; position: front+middle+back); (**o**) double-layer microsphere system; (**p**) bulk hydrogel system.

Figure 3 shows the influence of different drug release systems (Figure 3a,f) and microsphere features (Figure 3b–e) on GDNF volume fractions. The simulation time was 519 s with 9 s burst release and 510 s continuous release, and identical simulation time was applied to all the models to evaluate the GDNF volume fraction at the last time step. From Figure 3a, it can be found that double-layer microsphere (9.27 × 10^−10^) achieved the highest GDNF volume fraction, with a magnitude of roughly 1.8 and 3 times compared with the single-layer microsphere (4.98 × 10^−10^) and bulk hydrogel systems (3.34 × 10^−10^), respectively. Figure 3b–e reflect the effect of the microsphere features on GDNF volume fraction in a single-layer microsphere system. It can be seen that eight microspheres, 0.1 mm diameter, 0.15 mm adjacent distance and front-middle-back placement can achieve higher GDNF volume fraction from each group. Therefore, by combining those suggested settings, it is supposed that GDNF release performance can be raised to the next level. The combined model was evaluated again, and the corresponding results are shown in the last column of Figure 3f. According to Figure 3f, it can be seen that double-layer microsphere system still has the best performance among all the drug release systems. However, the increase between the default single-layer microsphere system and the combined model is over 60% by changing the microsphere features from multiple dimensions. Figure 4 shows the GDNF distribution among all the models at the last time step. 

### 3.3. GDNF Volume Fraction under the Assumption of Constant Growth Factor Mass

**Figure 5:**
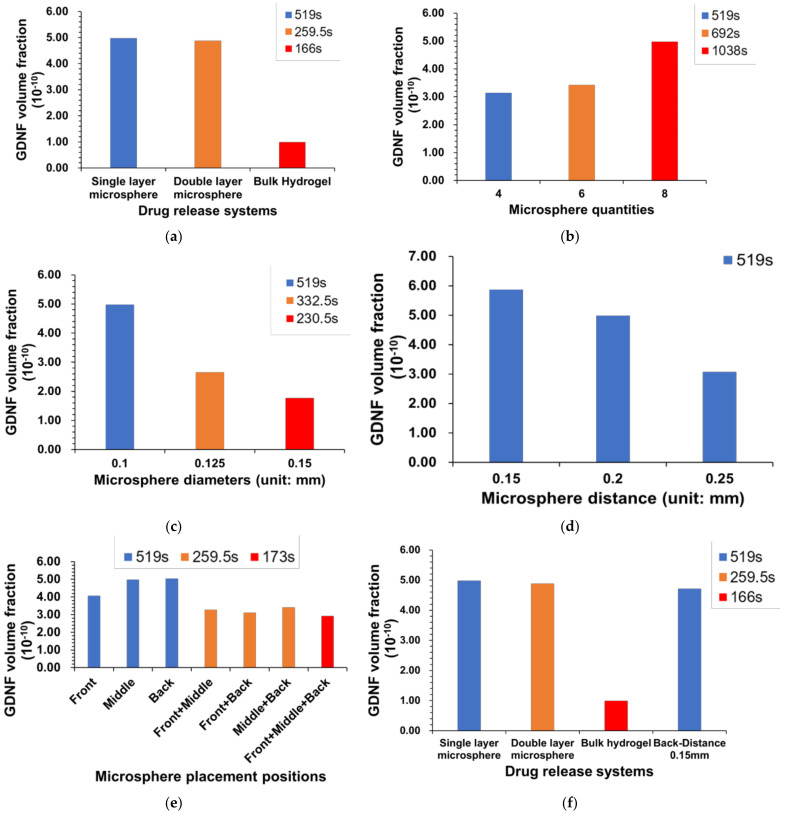
GDNF volume fraction of different drug release systems under the assumption of constant growth factor mass. (**a**) Comparison among single- and double-layer microsphere and bulk hydrogel systems. (**b**–**e**) Comparison within single-layer microsphere only. (**f**) Comparison between three drug release systems and the combined model.

**Figure 6:**
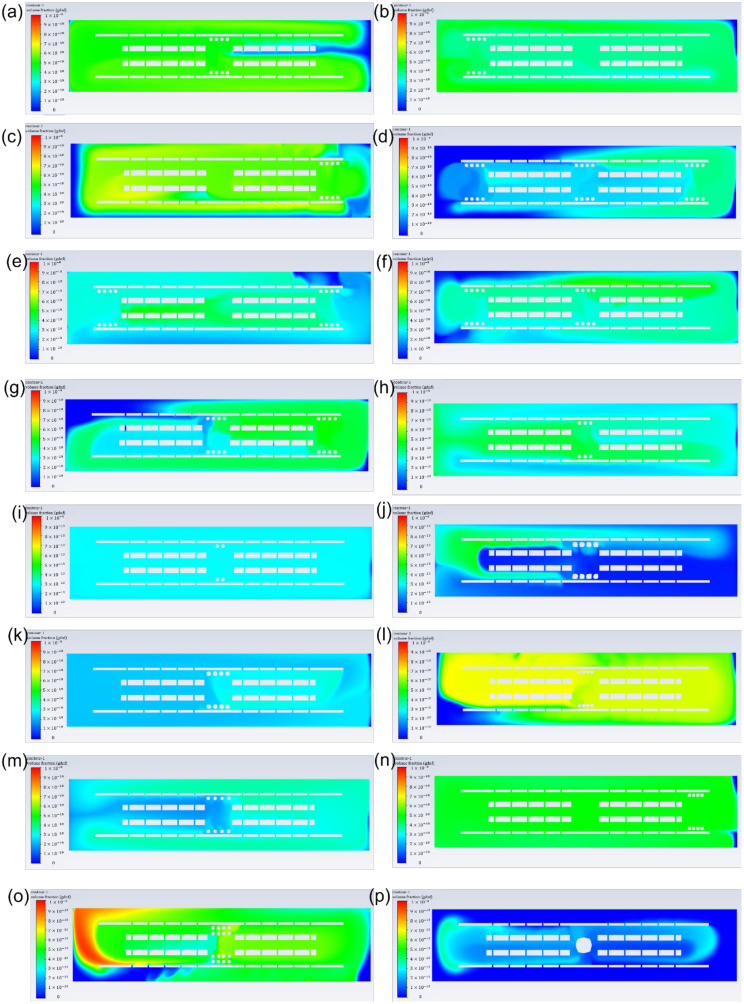
GDNF distribution of different drug release systems under the assumption of constant growth factor mass. (**a**) Single-layer microsphere system (microsphere features: quantity: 8; diameter: 0.1 mm; adjacent distance: 0.2 mm; position: middle); (**b**) single-layer microsphere system (microsphere features: quantity: 8; diameter: 0.1 mm; adjacent distance: 0.2 mm; position: front); (**c**) single-layer microsphere system (microsphere features: quantity: 8; diameter: 0.1 mm; adjacent distance: 0.2 mm; position: back); (**d**) single-layer microsphere system (microsphere features: quantity: 24; diameter: 0.1 mm; adjacent distance: 0.2 mm; position: front+middle+back); (**e**) single-layer microsphere system (microsphere features: quantity: 16; diameter: 0.1 mm; adjacent distance: 0.2 mm; position: front+back); (**f**) single-layer microsphere system (microsphere features: quantity: 16; diameter: 0.1 mm; adjacent distance: 0.2 mm; position: front+middle); (**g**) single-layer microsphere system (microsphere features: quantity: 16; diameter: 0.1 mm; adjacent distance: 0.2 mm; position: middle+back); (**h**) single-layer microsphere system (microsphere features: quantity: 6; diameter: 0.1 mm; adjacent distance: 0.2 mm; position: middle); (**i**) single-layer microsphere system (microsphere features: quantity: 4; diameter: 0.1 mm; adjacent distance: 0.2 mm; position: middle); (**j**) single-layer microsphere system (microsphere features: quantity: 8; diameter: 0.15 mm; adjacent distance: 0.2 mm; position: middle); (**k**) single-layer microsphere system (microsphere features: quantity: 8; diameter: 0.125 mm; adjacent distance: 0.2 mm; position: middle); (**l**) single-layer microsphere system (microsphere features: quantity: 8; diameter: 0.1 mm; adjacent distance: 0.15 mm; position: middle); (**m**) single-layer microsphere system (microsphere features: quantity: 8; diameter: 0.1 mm; adjacent distance: 0.25 mm; position: middle); (**n**) single-layer microsphere system (microsphere features: quantity: 8; diameter: 0.1 mm; adjacent distance: 0.15 mm; position: front+middle+back); (**o**) double-layer microsphere system; (**p**) bulk hydrogel system.

To maintain a constant growth factor, simulation time was adjusted based on the surface area of drug release system, which is illustrated in Table 2. GDNF volume fraction and distribution among different drug release systems are shown in Figure 5 and Figure 6, respectively. From Figure 5a, it can be seen that the single-layer microsphere system (4.98 × 10^−10^) and the double-layer microsphere system (4.88 × 10^−10^) have a comparable GDNF volume fraction, which is much higher than that of bulk hydrogel system (9.90 × 10^−11^). Figure 5b–e illustrate the effect of different microsphere features (quantity, diameter, adjacent distance, placement locations) on GDNF volume fraction in the single-layer microsphere system. Assuming the surface area remains constant when the distance between microspheres is changed, the simulation time of the models in Figure 5d is equal to 519 s, which is the default simulation time. From Figure 5b–e, it can be found that eight microspheres, 0.1 mm diameter, 0.15 mm distance, and back placement achieve the highest GDNF volume fraction in each experiment group, respectively. Therefore, Figure 5f was constructed by integrating those preferred settings into one drug release system, then calculating the GDNF release performance and comparing it with three predetermined drug release systems. From Figure 5f, it can be seen that the GDNF volume fraction can be slightly increased by the microsphere movement from middle to back and the denser arrangement of microspheres (0.15 mm adjacent distance). However, the combined model (4.71 × 10^−10^) integrating both back placement and 0.15 mm microsphere distance results in an even worse performance compared with solely adjusting the microsphere position to back and distance to 0.15 mm in Figure 5e,d, respectively. 

### 3.4. GDNF Volume Fraction among Different Hydrogel Films

**Figure 7:**
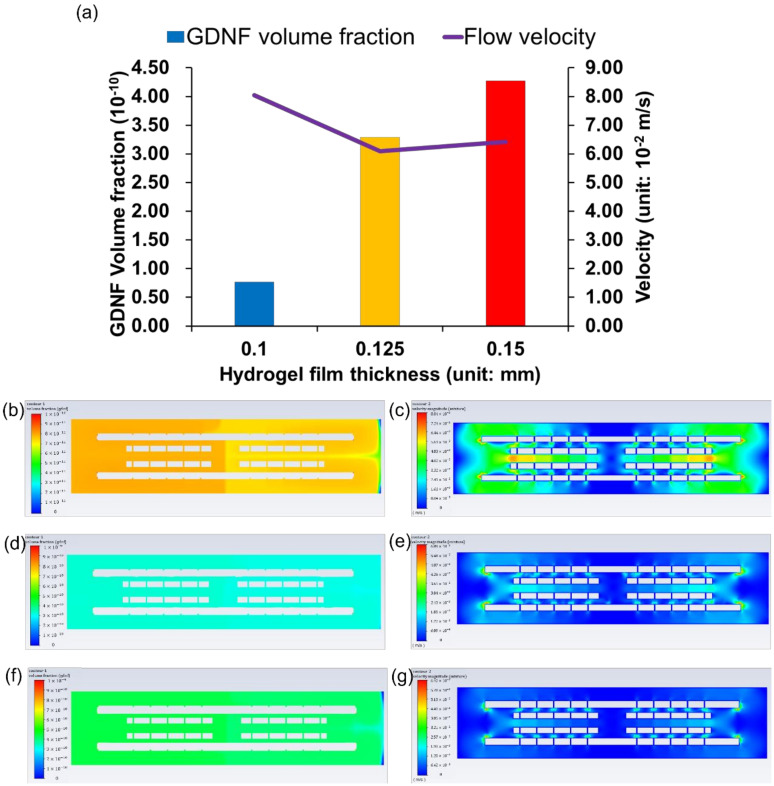
(**a**) GDNF volume fraction and flow velocity of hydrogel film systems with different film thicknesses. (**b**,**d**,**f**) GDNF distribution of three hydrogel film systems with film thicknesses from 0.1 mm to 0.15 mm. (**c**,**e**,**g**) Flow velocity of three hydrogel film systems with film thicknesses from 0.1 mm to 0.15 mm.

The influence of different hydrogel film thicknesses on GDNF volume fraction was examined and the results are shown in Figure 7a. A uniform simulation time of 519 s (9 s burst release and 510 s continuous release) was applied to all hydrogel film models. From Figure 7a, it can be seen that when the film thickness increased from 0.1 mm (7.69 × 10^−11^) to 0.125 mm (3.29 × 10^−10^), the GDNF volume fraction increased by 328%, while when the film thickness increased from 0.125 mm to 0.15 mm (4.28 × 10^−10^), the GDNF volume fraction increased by only 30%. Furthermore, the relationship between flow velocity and GDNF volume fraction is not monotonically increasing, which illustrates that higher velocity can not only facilitate the diffusion of growth factors, but can also accelerate the mixture fluid (including growth factor) flowing out of the structure. Figure 7b–g show the GDNF distribution and velocity magnitude of all the hydrogel film models. Similar GDNF distributions are found in all of the models with a gradually increasing GDNF volume fraction from 7.69 × 10^−11^ to 4.28 × 10^−10^. The highest velocity is achieved around the inner edges at both proximal and distal sides of the multichannel NGC for all the hydrogel films. 

## 4. Discussion

In most of the growth-factor-embedded scaffolds, microspheres and hydrogels are the two most commonly used drug carriers, owing to their well-developed fabrication method and controllable release profile. However, the variation of microsphere and hydrogel features is usually limited, leading to a lack of evaluation in the performance of different microsphere and hydrogel-based drug release systems. Therefore, in this study, the effect of different drug release systems on growth factor (GDNF) volume fraction in a multichannel NGC was carried out by controlling a constant simulation time and growth factor mass. Furthermore, growth-factor-embedded hydrogel films with different thicknesses were simulated to find out the most efficient model. 

Note that for both microsphere and hydrogel, the material should have proper biodegradability, because the growth factor is sealed inside and is supposed to be released along with the degradation process; thus, the release time of growth factors is usually determined by the degradability of the carrier materials. Furthermore, the release time is limited to less than 60 days in most cases; thereby, it is important to release and maintain a high growth factor concentration for a fixed release time. The total simulation time was scaled down from an existing GDNF release profile at the same proportions, and was 519 s composed of 9 s burst release and 510 s continuous release [19]. Figure 3 and Figure 4 show the magnitude and distribution of GDNF among different drug release systems at the last time step. The figures illustrate that the double-layer microsphere system achieves the highest GDNF volume fraction compared to the other two drug release systems. The huge increase can be attributed to there being twice as many microspheres, which provide more tunnels through which to deliver the growth factor into the scaffolds. In the single-layer microsphere system, the GDNF volume fraction was significantly changed by adjusting the microsphere features, including microsphere diameter, quantity, adjacent distance, and locations. The combination of smaller adjacent distance (0.15 mm) and multiple placements (front+middle+back) of microspheres improved the GDNF volume fraction by 67% from 4.98 × 10^−10^ to 8.3 × 10^−10^. Although more microspheres were implanted in the combined group than in the double-layer microsphere system, which was supposed to achieve a higher GDNF volume fraction, the double-layer microsphere system still possessed the best performance under a constant simulation time. The higher GDNF volume fraction of the double-layer microsphere system probably benefits from the small distance between the two layers, which could act as a flow accelerator to speed up the flow and deliver the growth factor more efficiently to the whole structure. Therefore, the result shows that quantity and adjacent distance (both vertically and horizontally) of microspheres have the maximum impact on the GDNF volume fraction, but other factors including microsphere diameter and locations could also influence the GDNF concentration. 

Since the NGC needs to connect with the original nerve in human body, the diameter of NGC and the human nerve must be the same. Thus, it can be inferred that tiny lumen size is achieved, therefore leading to a limited number of embedded growth factors in the NGC. The growth factor mass is controlled by the drug release system surface area and simulation (release) time. Higher surface area would result in shorter simulation time and vice versa. Figure 5 and Figure 6 show the GDNF magnitude and distribution in a variety of drug release systems. The double-layer microsphere system does not achieve a superior performance, as it assumes a constant simulation time, but has a GDNF volume fraction comparable to that of the single-layer microsphere system. For the single-layer microsphere system, decreasing the microsphere quantity and increasing the adjacent distance would cause a disadvantageous GDNF volume fraction, which is in agreement with the NGC groups under the assumption of constant simulation time. From Figure 5e, it can be seen that back position slightly improved the GDNF volume fraction compared to the middle position. However, the GDNF volume fraction of the combined model is lower than that before the combination, as shown in Figure 5f. Considering the even distribution of GDNF in Figure 6n, it is possible that the structure reaches the steady state between the GDNF and interstitial fluid before the end of the simulation, thereby allowing the excessive growth factor to flow out of the structure and decreasing the GDNF volume fraction. The reduced GDNF volume fraction of the combined model reveals that delaying the time to steady state of the drug release system is crucial to maintaining high growth factor concentrations. 

The effect of different hydrogel film thicknesses on the GDNF volume fraction was evaluated in this study, and the GDNF magnitude as well as distribution are shown in Figure 7. Please note that for hydrogel film systems, the surface area is more than 150 times higher than the other three drug release systems; therefore, it is meaningless to compare the GDNF volume fraction with other drug release systems. As can be seen from Figure 7, thicker hydrogel film can contribute to higher GDNF volume fraction, and similar GDNF and velocity distributions are found from all the hydrogel films. The poor volume fraction of GDNF in the 0.1 mm hydrogel film can be attributed to the high flow velocity, which accelerated the flow of GDNF out of the structure, but failed to efficiently transport and maintain GDNF in the whole structure, especially in the hydrogel film system. As the same parameter can lead to opposite effects in different drug release systems, it is particularly important to evaluate the growth factor release performance among various drug release systems. Other hydrogel properties, like swelling ratio, biodegradability, and viscoelasticity, can influence the drug release performance as well; therefore, it is important to study the influence of these hydrogel properties in future research. Furthermore, nanocomposite gels or hydrogel–nanoparticle combinations, as a novel approach, have the potential to integrate the advantages of both drug release systems, and therefore can be explored as a desired drug release system in the future [49]. 

Under the assumptions of constant simulation time and growth factor mass, double-layer microsphere system possesses excellent GDNF release performance, which can be used as an ideal drug release system for multichannel NGC. However, the thickness of double-layer microsphere systems (>200 μm) could take up more than half of the channel size (340 μm), resulting in the potential risk of blocking the peripheral channels of the multichannel NGC. Thus, in the future, it is necessary to carefully balance the thickness of the drug release system and channel diameter. Due to the easy implantation of hydrogel film and the even distribution of GDNF throughout the whole structure (which is crucial to guide the nerve regeneration at the same rate), hydrogel film systems can be regarded as a strong candidate in drug release systems. 

## 5. Conclusions

This study used the CFD simulation approach to examine the growth factor diffusion process in drug release system embedded multichannel NGCs. Two commonly used carriers and four different drug release systems, including a single-layer microsphere system, a double-layer microsphere system, bulk hydrogel, and hydrogel film, were chosen, and the effect of microsphere features and hydrogel thicknesses on the GDNF volume fraction and distribution were evaluated. Under the assumptions of constant simulation time and constant growth factor mass, the double-layer microsphere system achieves excellent GDNF release performance, and therefore it can be regarded as the best choice among all the drug release systems. However, the ratio of channel diameter to the thickness of the double-layer microsphere system needs to be carefully balanced before integrating the drug release system; otherwise, the double-layer microsphere system might block the peripheral channels and hinder the diffusion of growth factors. For the single-layer microsphere system, the combination of smaller adjacent distance (0.15 mm) and multiple positions (front+middle+back) of microspheres improved the GDNF volume fraction by 67% under the assumption of constant simulation time. Although the GDNF volume fraction can be increased by moving the microspheres from middle to back and decreasing the adjacent distance from 0.2 mm to 0.15 mm under the assumption of constant growth factor mass, the combined model quickly reached the steady state owing to the rapid flow velocity, which leads to the decrease of GDNF volume fraction of the combined model. Therefore, it is important to prolong the time for the drug release system to reach steady state to maintain a high GDNF concentration. Furthermore, GDNF volume fraction increased monotonously with the increase of the thickness of hydrogel film, and hydrogel film with 0.15 mm thickness achieves the best performance without blocking the channels. This study can be treated as a guideline for the design and application of drug release systems in multichannel NGC. Further research can be carried out by investigating different NGC types and growth factor types to more comprehensively summarize the application of drug release systems in NGC. 

## Figures and Tables

**Table 1:** Illustration of how to narrow down the growth factor release profile.

	Total Release Time (s)	Burst Release Mass (ng)	Burst Release Time (s)	Burst Release Velocity (ng/s)	Continuous Release Mass (ng)	Continuous Release Time (s)	Continuous Release Velocity (ng/s)
Original release profile	5,184,000	5	86,400	5.79 × 10^−5^	1.5	5,097,600	2.94 × 10^−7^
Modified release profile	519	0.0005	9	5.56 × 10^−5^	0.00015	510	2.94 × 10^−7^

**Table 2:** Simulation time of the various drug release systems.

Drug Release Systems	Microsphere Diameter (mm)	Microsphere Quantity	Microsphere Placement	Surface Area (mm^2^)	Burst Release Time (s)	Continuous Release Time (s)	Total Simulation Time (s)
Single-layer microsphere	0.1	8	Front/middle/back	0.251	9	510	519
Single-layer microsphere	0.1	16	Front+middle/front+back/middle+back	0.502	4.5	255	259.5
Single-layer microsphere	0.1	24	Front+middle+back	0.753	3	170	173
Single-layer microsphere	0.125	8	Middle	0.393	6	326.5	332.5
Single-layer microsphere	0.15	8	Middle	0.565	4	226.5	230.5
Single-layer microsphere	0.1	6	Middle	0.188	12	680	692
Single-layer microsphere	0.1	4	Middle	0.126	18	1020	1038
Double-layer microsphere	0.1	16	Middle	0.502	4.5	255	259.5
Bulk hydrogel	0.5	1	Middle	0.785	3	163	166

## Data Availability

All data are provided in the manuscript.

## References

[1] Cinteza D., Persinaru I., Maciuceanu Zarnescu B.M., Ionescu D., Lascar I. (2015). Peripheral Nerve Regeneration—An Appraisal of the Current Treatment Options. Maedica.

[2] Sedaghati T., Jell G., Seifalian A.M., Orlando G., Lerut J., Soker S., Stratta R.J. (2014). Chapter 57—Nerve Regeneration and Bioengineering. Regenerative Medicine Applications in Organ Transplantation.

[3] Dietzmeyer N., Förthmann M., Grothe C., Haastert-Talini K. (2020). Modification of tubular chitosan-based peripheral nerve implants: Applications for simple or more complex approaches. Neural Regen. Res..

[4] Hood B., Levene H.B., Levi A.D. (2009). Transplantation of autologous Schwann cells for the repair of segmental peripheral nerve defects. Neurosurg. Focus.

[5] Cinal H., Barin E.Z., Kara M., Karaduman H., Cengiz I.Z., Tan O., Demirci E. (2020). A new method to harvest the sural nerve graft. Eurasian J. Med..

[6] Zarrintaj P., Zangene E., Manouchehri S., Amirabad L.M., Baheiraei N., Hadjighasem M.R., Farokhi M., Ganjali M.R., Walker B.W., Saeb M.R. (2020). Conductive biomaterials as nerve conduits: Recent advances and future challenges. Appl. Mater. Today.

[7] Gupta S., Mohapatra D., Chittoria R., Subbarayan E., Reddy S., Chavan V., Aggarwal A., Reddy L. (2019). Human skin allograft: Is it a viable option in management of burn patients?. J. Cutan. Aesthet. Surg..

[8] Zhang S., Vijayavenkataraman S., Chong G.L., Fuh J.Y.H., Lu W.F. (2019). Computational Design and Optimization of Nerve Guidance Conduits for Improved Mechanical Properties and Permeability. J. Biomech. Eng..

[9] Spencer A.R., Shirzaei Sani E., Soucy J.R., Corbet C.C., Primbetova A., Koppes R.A., Annabi N. (2019). Bioprinting of a Cell-Laden Conductive Hydrogel Composite. ACS Appl. Mater. Interfaces.

[10] Fadia N.B., Bliley J.M., DiBernardo G.A., Crammond D.J., Schilling B.K., Sivak W.N., Spiess A.M., Washington K.M., Waldner M., Liao H.T. (2020). Long-gap peripheral nerve repair through sustained release of a neurotrophic factor in nonhuman primates. Sci. Transl. Med..

[11] Xu P., Rosen K.M., Hedstrom K., Rey O., Guha S., Hart C., Corfas G. (2013). Nerve injury induces glial cell line-derived neurotrophic factor (GDNF) expression in schwann cells through purinergic signaling and the PKC-PKD pathway. GLIA.

[12] Höke A., Gordon T., Zochodne D.W., Sulaiman O.A.R. (2002). A decline in glial cell-line-derived neurotrophic factor expression is associated with impaired regeneration after long-term Schwann cell denervation. Exp. Neurol..

[13] Troullinaki M., Alexaki V.-I., Mitroulis I., Witt A., Klotzsche–von Ameln A., Chung K.-J., Chavakis T., Economopoulou M. (2019). Nerve growth factor regulates endothelial cell survival and pathological retinal angiogenesis. J. Cell. Mol. Med..

[14] Vijayavenkataraman S. (2020). Nerve guide conduits for peripheral nerve injury repair: A review on design, materials and fabrication methods. Acta Biomater..

[15] Arslantunali D., Dursun T., Yucel D., Hasirci N., Hasirci V. (2014). Peripheral nerve conduits: Technology update. Med. Devices Evid. Res..

[16] Zhou X., Quann E., Gallicano G.I. (2003). Differentiation of nonbeating embryonic stem cells into beating cardiomyocytes is dependent on downregulation of PKCβ and ζ in concert with upregulation of PKCε. Dev. Biol..

[17] Kokai L.E., Bourbeau D., Weber D., McAtee J., Marra K.G. (2011). Sustained growth factor delivery promotes axonal regeneration in long gap peripheral nerve repair. Tissue Eng. Part A.

[18] Silva A.K.A., Richard C., Bessodes M., Scherman D., Merten O.W. (2009). Growth factor delivery approaches in hydrogels. Biomacromolecules.

[19] Kokai L.E., Ghaznavi A.M., Marra K.G. (2010). Incorporation of double-walled microspheres into polymer nerve guides for the sustained delivery of glial cell line-derived neurotrophic factor. Biomaterials.

[20] Li J., Mooney D.J. (2016). Designing hydrogels for controlled drug delivery. Nat. Rev. Mater..

[21] Sheth S., Barnard E., Hyatt B., Rathinam M., Zustiak S.P. (2019). Predicting Drug Release From Degradable Hydrogels Using Fluorescence Correlation Spectroscopy and Mathematical Modeling. Front. Bioeng. Biotechnol..

[22] Carbinatto F.M., De Castro A.D., Evangelista R.C., Cury B.S.F. (2014). Insights into the swelling process and drug release mechanisms from cross-linked pectin/high amylose starch matrices. Asian J. Pharm. Sci..

[23] Li J., Wu C., Chu P.K., Gelinsky M. (2020). 3D printing of hydrogels: Rational design strategies and emerging biomedical applications. Mater. Sci. Eng. R Rep..

[24] Vijayavenkataraman S., Yan W.-C., Lu W.F., Wang C.-H., Fuh J.Y.H. (2018). 3D bioprinting of tissues and organs for regenerative medicine. Adv. Drug Deliv. Rev..

[25] Zhou J., Vijayavenkataraman S. (2021). 3D-printable conductive materials for tissue engineering and biomedical applications. Bioprinting.

[26] Soman S.S., Vijayavenkataraman S. (2020). Perspectives on 3d bioprinting of peripheral nerve conduits. Int. J. Mol. Sci..

[27] Wang Z., Wang Z., Lu W.W., Zhen W., Yang D., Peng S. (2017). Novel biomaterial strategies for controlled growth factor delivery for biomedical applications. NPG Asia Mater..

[28] Murphy W.L., Peters M.C., Kohn D.H., Mooney D.J. (2000). Sustained release of vascular endothelial growth factor from mineralized poly(lactide-co-glycolide) scaffolds for tissue engineering. Biomaterials.

[29] Cho H.J., Madhurakkat Perikamana S.K., Lee J.H., Lee J., Lee K.M., Shin C.S., Shin H. (2014). Effective immobilization of BMP-2 mediated by polydopamine coating on biodegradable nanofibers for enhanced in vivo bone formation. ACS Appl. Mater. Interfaces.

[30] Koffler J., Zhu W., Qu X., Platoshyn O., Dulin J.N., Brock J., Graham L., Lu P., Sakamoto J., Marsala M. (2019). Biomimetic 3D-printed scaffolds for spinal cord injury repair. Nat. Med..

[31] Yao L., Daly W., Newland B., Yao S., Wang W., Chen B.K.K., Madigan N., Windebank A., Pandit A. (2013). Improved axonal regeneration of transected spinal cord mediated by multichannel collagen conduits functionalized with neurotrophin-3 gene. Gene Ther..

[32] Pawelec K.M., Koffler J., Shahriari D., Galvan A., Tuszynski M.H., Sakamoto J. (2018). Microstructure and in vivo characterization of multi-channel nerve guidance scaffolds. Biomed. Mater..

[33] Zhao X., Fan C., Wang J., Xiong H., Zhu T., Liu Y., Pan H., Weijia Lu W. (2020). Bioinspired multichannel nerve guidance conduit based on shape memory nanofibers for potential application in peripheral nerve repair. ACS Nano.

[34] Yao L., Billiar K.L., Windebank A.J., Pandit A. (2010). Multichanneled collagen conduits for peripheral nerve regeneration: Design, fabrication, and characterization. Tissue Eng. Part C Methods.

[35] Gustafson K.J., Grinberg Y., Joseph S., Triolo R.J. (2012). Human distal sciatic nerve fascicular anatomy: Implications for ankle control using nerve-cuff electrodes. J. Rehabil. Res. Dev..

[36] Xu H., Zhang L., Bao Y., Yan X., Yin Y., Li Y., Wang X., Huang Z., Xu P. (2017). Preparation and characterization of injectable chitosan-hyaluronic acid hydrogels for nerve growth factor sustained release. J. Bioact. Compat. Polym..

[37] Wang P., Berry D., Moran A., He F., Tam T., Chen L., Chen S. (2020). Controlled Growth Factor Release in 3D-Printed Hydrogels. Adv. Healthc. Mater..

[38] Trujillo S., Gonzalez-Garcia C., Rico P., Reid A., Windmill J., Dalby M.J., Salmeron-Sanchez M. (2020). Engineered 3D hydrogels with full-length fibronectin that sequester and present growth factors. Biomaterials.

[39] Kim H., Kong W.H., Seong K.Y., Sung D.K., Jeong H., Kim J.K., Yang S.Y., Hahn S.K. (2016). Hyaluronate—Epidermal Growth Factor Conjugate for Skin Wound Healing and Regeneration. Biomacromolecules.

[40] Hong J.P., Kim Y.W., Lee S.K., Kim S.H., Min K.H. (2008). The effect of continuous release of recombinant human epidermal growth factor (rh-EGF) in chitosan film on full thickness excisional porcine wounds. Ann. Plast. Surg..

[41] Gil E.S., Panilaitis B., Bellas E., Kaplan D.L. (2013). Functionalized Silk Biomaterials for Wound Healing. Adv. Healthc. Mater..

[42] Zhang L., Ji R., Fu Y., Qi H., Kong F., Li H., Tangwarodomnukun V. (2020). Investigation on particle motions and resultant impact erosion on quartz crystals by the micro-particle laden waterjet and airjet. Powder Technol..

[43] Lackington W.A., Kočí Z., Alekseeva T., Hibbitts A.J., Kneafsey S.L., Chen G., O’Brien F.J. (2019). Controlling the dose-dependent, synergistic and temporal effects of NGF and GDNF by encapsulation in PLGA microparticles for use in nerve guidance conduits for the repair of large peripheral nerve defects. J. Control. Release.

[44] Yao W., Shen Z., Ding G. (2013). Simulation of interstitial fluid flow in ligaments: Comparison among Stokes, Brinkman and Darcy models. Int. J. Biol. Sci..

[45] Arora J., Hickey J.M., Majumdar R., Esfandiary R., Bishop S.M., Samra H.S., Middaugh C.R., Weis D.D., Volkin D.B. (2015). Hydrogen exchange mass spectrometry reveals protein interfaces and distant dynamic coupling effects during the reversible self-association of an IgG1 monoclonal antibody. mAbs.

[46] Yao W., Li Y., Ding G. (2012). Interstitial fluid flow: The mechanical environment of cells and foundation of meridians. Evid.-Based Complement. Altern. Med..

[47] Fischer H., Polikarpov I., Craievich A.F. (2004). Average protein density is a molecular-weight-dependent function. Protein Sci..

[48] Rodríguez F.J., Verdú E., Ceballos D., Navarro X. (2000). Nerve guides seeded with autologous Schwann cells improve nerve regeneration. Exp. Neurol..

[49] Giordano C., Albani D., Gloria A., Tunesi M., Rodilossi S., Russo T., Forloni G., Ambrosio L., Cigada A. (2011). Nanocomposites for Neurodegenerative Diseases: Hydrogel-Nanoparticle Combinations for a Challenging Drug Delivery. Int. J. Artif. Organs.

